# Comparison of the Vietnamese Diet with Diets from Other Countries for Sarcopenia

**DOI:** 10.21315/mjms2023.30.4.18

**Published:** 2023-08-24

**Authors:** Le Pham Tan Quoc, Nguyen Thi Bich Ngoc

**Affiliations:** Institute of Biotechnology and Food Technology, Industrial University of Ho Chi Minh City, Ho Chi Minh City, Vietnam

Dear Editor,

We have read the review article by Nazri et al. (1) with great interest. The authors showed the association between dietary patterns/food groups and sarcopenia. As we know, proper nutrition is essential to human health and can prevent certain diseases. The Mediterranean diet (MedDiet) is believed to be healthy and has an ameliorating effect on sarcopenia. This diet tends to include more plant-based foods, fruits and vegetables and uses olive oil as the primary source of fat. In particular, it comprises less red meat and other processed foods and includes alcohol in moderation with meals (2). A diet rich in fruits and vegetables, particularly those rich in antioxidants to reduce inflammation as well as oxidative stress, reduces the risk of sarcopenia in the elderly. This is completely suitable for the Vietnamese dietary pattern; the fertile land is ideal for growing food crops and vegetable sources of carbohydrates, which provide many vitamins and minerals as well as highly bioactive compounds. Light to moderate alcohol consumption may have beneficial effects on adult bone, leading to higher bone mineral density and reduced age-related bone loss (3). However, in Vietnam, hard alcohol or beer use is especially prevalent. The excessive consumption of alcohol increases the risk of osteoporosis (4). For the elderly, there is a strict association between sarcopenia and bone density (5). In our opinion, the MedDiet has many features in common with the Vietnamese diet, but hard alcohol should be limited and may be substituted with fermented juice or wine.

In addition, the Western diet mainly comprises high amounts of fat from butter and protein from red meats. Also, potatoes, high-sugar desserts and sweets are consumed. This diet provides a high amount of energy from red meat and sweets, which can prevent an energy deficiency in people with sarcopenia. However, the excessive consumption of red meat and sweets is associated with cardiovascular disease, diabetes and different cancers. Therefore, Rondanelli et al. (6) recommended consuming red meat less than two times/week for the elderly to prevent sarcopenia. Today, many countries have also reduced their consumption of red meat because of the environmental pollution caused by its production. Some regions have developed artificial meat from bacteria, which is sure to be a novel future food trend. In Vietnam, pork or chicken is commonly consumed due to the affordable price and high nutritional value. White meat also provides dietary protein intake and is used as a meal supplement to improve muscle mass. It seems to lower cardiovascular disease risk more readily than red meat (beef) (7). In addition, the need to import most of the beef consumed in Vietnam has made it a food for high-income earners. Until now, white meat is still an optimum choice for most Vietnamese.

The Japanese diet is another highly nutritious diet, comprising a variety of foods derived from both animals and plants. One of the key components of the Japanese diet is fish, a rich source of animal protein that is also low in saturated fat and high in omega-3 fatty acids and vitamin D, which improves muscle mass, performance and physical fitness in the elderly (8). Adherence to the traditional Japanese diet is also associated with a low rate of sarcopenia in the elderly (9). In Vietnam, marine and river resources are readily available, so it is convenient to consume fish as a staple food in the same way as the Japanese.

In short, we should selectively adopt the favourable culinary trends of various regions; doing so will bring many health benefits and provide a reasonable diet for individuals with sarcopenia, thereby helping them to regain their physical health and mental well-being. Based on the perspectives mentioned above, Mediterranean or Japanese dietary patterns should be adopted in Vietnam to lower the risk of sarcopenia.
